# Brucella-infected abdominal aortic aneurysm: management strategies for an uncommon aneurysm

**DOI:** 10.3389/fmed.2023.1271217

**Published:** 2023-11-02

**Authors:** Huibo Ma, Yuling Yang, Huanhuan Liu, Xiaozhi Sun, Yongxin Li, Mingjin Guo

**Affiliations:** ^1^Department of Vascular Surgery, Affiliated Hospital of Qingdao University, Qingdao, Shandong, China; ^2^Department of Infectious Diseases, Affiliated Hospital of Qingdao University, Qingdao, Shandong, China; ^3^Department of General Surgery, Affiliated Yantai Yuhuangding Hospital of Qingdao University, Yantai, Shandong, China

**Keywords:** brucella-IAAA, open surgery, EVAR, imaging examination, doxycycline

## Abstract

**Objective:**

The occurrence of Brucella-induced abdominal aortic aneurysms is an exceedingly rare phenomenon, yet it stands as one of the most severe complications within this context. The combined utilization of serological testing and imaging diagnostics has been validated as an effective approach for the identification of Brucella-induced abdominal aortic aneurysms. Presently, the predominant therapeutic strategies encompass antibiotic treatment and surgical intervention. Nonetheless, ongoing controversies persist concerning the establishment of diagnostic criteria, the optimal timing and selection of antibiotic regimens, and the nuanced decision between open surgical procedures and endovascular interventions. Through a meticulous analysis of cases originating from our institution as well as a comprehensive review of previously documented instances, we aim to engage in a detailed discourse on the salient diagnostic and therapeutic facets surrounding Brucella-induced abdominal aortic aneurysms.

**Methods:**

We conducted a retrospective summary of three cases involving Brucella-induced abdominal aortic aneurysms treated within our institution. Furthermore, we performed a comprehensive PubMed search, without imposing restrictions on language or publication year, to identify pertinent literature pertaining to Brucella-induced abdominal aortic aneurysms. The selection criteria primarily focused on case reports delineating occurrences of abdominal aortic aneurysms attributed to Brucella infection.

**Results:**

We present three distinct cases of Brucella-induced abdominal aortic aneurysms managed at our institution, providing comprehensive insights into the employed diagnostic and therapeutic approaches. Additionally, over the past five decades, a total of 24 cases in 23 publications of Brucella-induced abdominal aortic aneurysms have been reported on PubMed. The earliest report dates back to 1976.

**Conclusion:**

Our analysis suggests that Brucella-induced abdominal aortic aneurysm is characterized by a remarkably low incidence but is associated with a substantial risk of life-threatening complications. The integration of serological and imaging assessments assumes pivotal importance in facilitating prompt diagnosis of this condition. The prompt initiation of targeted antibiotic therapy is recommended, and the selection of appropriate surgical strategies should be guided by considerations including aneurysm dimensions and morphological attributes. The timely identification and intervention carry utmost significance in retarding disease advancement and ameliorating unfavorable clinical outcomes.

## 1. Introduction

Infected abdominal aortic aneurysm (IAAA) arises as a consequence of direct or indirect infection of the abdominal aorta by various pathogenic microorganisms (1). This infectious process can lead to detrimental effects on the blood vessel walls, ultimately resulting in aneurysmal dilatation (2). Despite its rarity, IAAA represents a formidable and life-threatening condition characterized by an abrupt onset, advanced disease stage, rapid progression, and an unfavorable prognosis (3). Among the most frequently encountered pathogens associated with IAAA, Salmonella and *Staphylococcus aureus* hold prominence (4, 5). Certainly, while the likelihood of infection by other bacteria is relatively low, sporadic reports have been documented.

Brucellosis, caused by the bacteria of the genus Brucella, represents the most prevalent bacterial zoonosis globally (6). It is endemic in the Mediterranean, Middle East countries, and Latin America (7, 8). The primary routes of human infection include the ingestion of unpasteurized dairy products, inhalation of aerosolized infectious particles, and direct contact with infected animals (9, 10). Herders, veterinarians, and slaughterhouse workers are considered high-risk occupations for acquiring this disease (11, 12). Brucella can replicate within various mammalian cells and primarily affects the musculoskeletal, respiratory, digestive, urinary, and reproductive systems (13–15). In the cardiovascular system, the incidence of Brucella infection is extremely low, accounting for approximately 3%. Moreover, the abdominal aorta is not traditionally considered a common site for Brucella infection (16).

In recent years, it has been discovered that the incidence of Brucella-infected abdominal aortic aneurysms (Brucella-IAAA) remains exceedingly low when inexperienced diagnostic strategies and unclear understanding are employed (17). In this Grand Round, we show a series of 3 compelling cases involving patients afflicted by Brucella-IAAA. Notably, these individuals received non-open surgical treatment modalities, which resulted in favorable prognoses. Concurrently, we conduct a comprehensive review of published literature to elucidate the clinical manifestations, diagnostic strategies, prognostic indicators, and therapeutic interventions pertaining to Brucella-IAAA.

## 2. Methods

### 2.1. Case report

We conducted a retrospective analysis of three cases of abdominal aortic aneurysm (AAA) patients diagnosed with Brucella infection and treated at our medical center from 2021 to 2023. The analysis involved a comprehensive review of their medical histories, presenting symptoms, imaging findings, serological data, treatment protocols, and prognostic outcomes. Cases of other aortic diseases attributed to Brucella, such as thoracic aortic aneurysms, were excluded from the study. This research received approval from the Institutional Review Board of Qingdao University Affiliated Hospital, and informed consent was obtained from all patients and their legal guardians.

### 2.2. Literature review

A PubMed search was conducted in June 2023 without language or year of publication restrictions to identify articles related to AAA (abdominal aortic aneurysm) associated with Brucella by us. The search strategy adhered to the Preferred Reporting Items for Systematic Reviews and Meta-Analyses (PRISMA) guidelines, combining the terms “Brucella” and “aneurysm” to focus on the AAA-related subset. Additionally, we manually screened the reference lists of included articles to identify other relevant studies. Eligible for inclusion were case reports or series describing Brucella-induced infected (pseudo) aneurysms. Cases involving peripheral or cerebral arteries, thoracic aorta, isolated aortic valve endocarditis, or dual reports were excluded. Two authors (HL and XS) independently reviewed all identified articles for their relevance.

## 3. Cases and results

### 3.1. Patient 1

A 59-year-old male was referred to our institution presenting with a one-month history of nausea, vomiting, bilateral lower limb swelling, pain, and tightness. The patient denied experiencing fever, night sweats, or weight loss. He had a history of poor general health and previously abnormal liver function test results. On examination, a palpable, firm mass measuring 20 cm × 15 cm was detected in the right lower abdomen quadrant. Computed Tomography Angiography (CTA) of the aorta revealed an unstable pseudoaneurysm in the right common iliac artery, characterized by uneven density, as well as a rough and thickened wall. Blood analysis revealed an elevated level of gamma-glutamyl transferase (265.4 U/L), along with increased levels of C-reactive protein (CRP) (29.13 mg/L) and D-dimer (1,300 ng/mL). A diagnosis of a ruptured iliac artery aneurysm was established, and surgical intervention was scheduled for the following day.

However, the patient experienced a sudden onset of high fever with a body temperature exceeding 40°C, accompanied by intensified right lower abdominal pain that night. The patient reported having experienced several episodes of unexplained fever in the past year, which resolved spontaneously without specific treatment. Additionally, the patient disclosed a history of privately slaughtering and cooking sheep. Laboratory tests for infectious diseases were conducted, confirming positive results for the Rose Bengal plate agglutination test (RBPT) and standard tube agglutination test (SAT), with a SAT titer of 1:400. Furthermore, blood cultures yielded the growth of *Brucella melitensis*.

**Figure 1:**
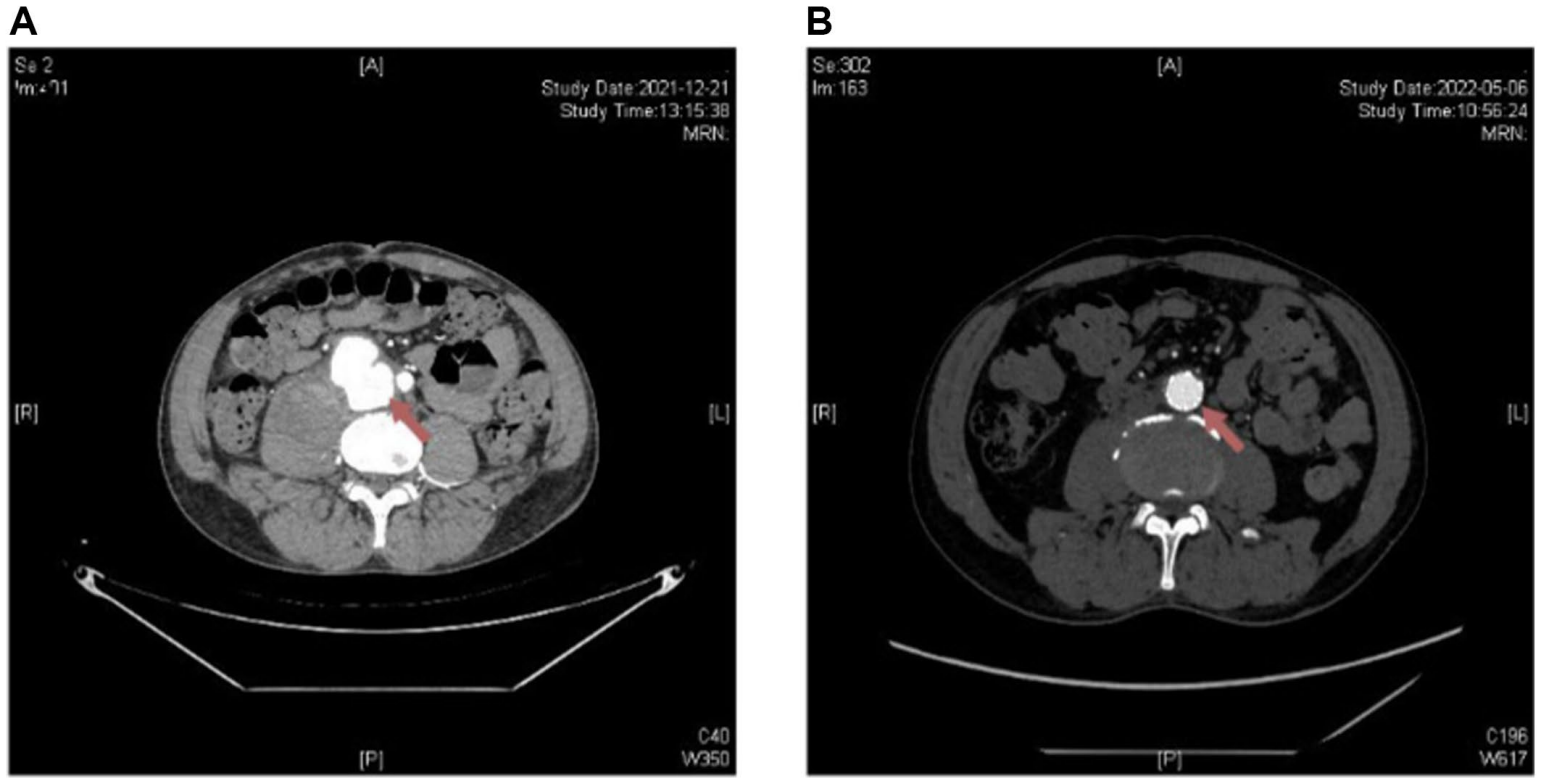
Radiological changes on abdominal aortic computed tomography angiography (CTA) were observed in Patient 1 before and after treatment. **(A)** Upon admission: the density of the abdominal aorta and right iliac artery is uneven, with wall thickening and roughness, and the fat planes appear indistinct. **(B)** After treatment: the walls of the abdominal aorta and right iliac artery show slight thickening, with significant improvement compared to the previous examination.

Based on the updated understanding of the disease, the treatment plan was modified to incorporate triple therapy, including oral rifampicin (600 mg, once daily), minocycline (100 mg, twice daily), and intravenous ceftriaxone sodium (2000 mg, twice daily). After 8 days, the infection symptoms were effectively controlled. Subsequently, the patient underwent endovascular stent graft repair of the abdominal aortic aneurysm (AAA), as previously planned. The patient continued to receive active triple therapy and exhibited a satisfactory recovery following surgery. He was discharged on the fifth postoperative day, as the fever and abdominal pain had completely resolved. The patient completed a total of 6 months of Brucella therapy at the local hospital. During the 6-month follow-up period after surgery, he remained free of abdominal pain recurrence. Repeat abdominal aorta CTA demonstrated nearly complete resolution of the patch shadows around the abdominal aorta (Figure 1).

### 3.2. Patient 2

A 64-year-old male was admitted to the hospital due to intermittent lower abdominal pain lasting over a month, which worsened at night. The patient denied having a history of hypertension, diabetes mellitus, or any infectious disease. Additionally, he reported no residence or travel history to epidemic areas. CTA of the aorta revealed a false aneurysm in the left infrarenal aorta, surrounded by nodular lesions with uneven density and multiple patchy shadows. Laboratory tests showed positive results for antinuclear antibody, anti-ribonucleoprotein antibody, and anti-Sm antibody. However, the patient exhibited no other positive signs associated with connective tissue diseases, such as cold-induced hand discoloration or frequent oral ulcers. Other immune-related indicators were negative, effectively ruling out connective tissue diseases. With a definitive diagnosis of AAA, the patient underwent endovascular stent graft repair of the infrarenal AAA, leading to immediate relief of abdominal pain. The patient was discharged 5 days after the procedure.

**Figure 2:**
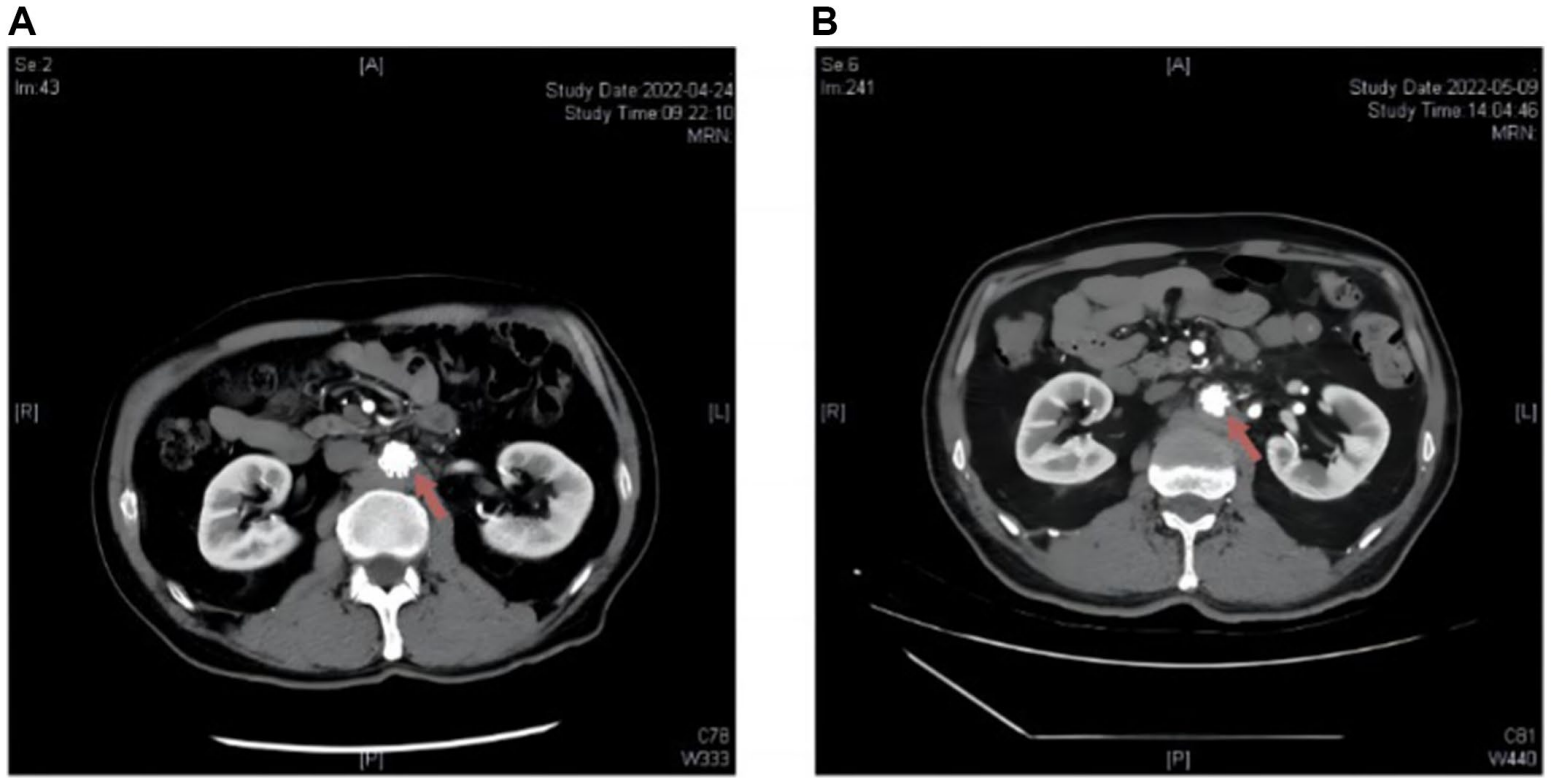
Radiological alterations on abdominal aortic CTA before and subsequent to antibiotic administration in Patient 2 post endovascular aneurysm repair (EVAR). **(A)** Upon admission: multiple patchy and nodular abnormal density shadows around the abdominal aorta. **(B)** After treatment: the abnormal density shadows around the abdominal aorta decreased compared to the previous findings.

Unfortunately, on the third day after discharge, the patient experienced a sudden onset of fever, with temperatures exceeding 39°C. Although he attempted to alleviate the symptoms using Ibuprofen, the effect was not significant. Additionally, he experienced recurrent periumbilical dull pain. Eight days later, the patient was readmitted for further treatment. Repeat CTA revealed a significant increase in the number of nodular lesions with uneven density and patchy shadows around the abdominal aorta compared to the previous examination. Laboratory test results showed multiple abnormalities, including elevated CRP levels (31.3 mg/L) and an increased erythrocyte sedimentation rate (ESR) (25.3 mm/1 h). Moreover, the antinuclear antibody, anti-ribonucleoprotein antibody, and anti-Sm antibody remained positive. Importantly, further examination revealed positive results for the RBPT and SAT, with a SAT titer of 1:50. A detailed inquiry into the patient’s personal history unveiled that his residence was in close proximity to a sheep pasture, posing a high risk of exposure to sheep manure. Although blood cultures showed no growth of microorganisms, the patient was diagnosed with Brucella infection based on imaging data, laboratory examinations, and clinical symptoms. Following the definitive diagnosis, the patient received appropriate medication, including oral rifampicin (600 mg, once daily), minocycline (100 mg, twice daily), and levofloxacin (750 mg, twice daily). Within one week, the fever and abdominal discomfort completely resolved, leading to the patient’s request for discharge. He continued to receive standard treatment for eight weeks at the local infectious disease hospital. Subsequent CTA revealed a significant reduction in the size of the AAA, with the disappearance of shadows surrounding the aneurysm at the 1-month follow-up (Figure 2).

### 3.3. Patient 3

A 60-year-old male patient, known to have coronary heart disease for over eight years, presented with persistent needle-like left lower abdominal pain lasting approximately ten days. The patient denied having a history of fever, arthralgia, rash, weight loss, or other systemic symptoms recently. He worked as a farmer and had several goats under his care. Upon abdominal examination, a painful, palpable, and pulsatile mass was detected in the left lower abdomen, with no signs of peritonitis. CTA revealed a 3.8-cm infrarenal AAA surrounded by inflammatory changes in the adjacent soft tissue. Furthermore, Positron Emission Tomography-CT examination exhibited soft tissue masses in the anterior and lateral aspects of the lower abdominal aorta, demonstrating significantly increased metabolic activity with a maximum standardized uptake value of approximately 19.3, suggestive of periarteritis. Laboratory investigations ruled out abnormalities in routine blood indicators, as well as liver and kidney function. However, the CRP level was markedly elevated at 6.62 mg/L. Additionally, ESR was elevated (>20 mm/h), accompanied by a total white blood cell count in peripheral blood exceeding 1 × 10^8 /mL. Considering the aforementioned clinical features, laboratory findings, and CTA results, the differential diagnosis strongly indicated an infectious aneurysm. Further tests, excluding indicators such as antistreptolysin O, antinuclear antibodies, and the quantifier test for tuberculosis, revealed a positive result in the RBPT. Subsequent SAT also yielded a positive result with a titer of 1:400. Both tests are specific for detecting Brucella infection. Although blood cultures for pathogens remained consistently negative, the diagnosis of Brucella-IAAA was established.

**Figure 3:**
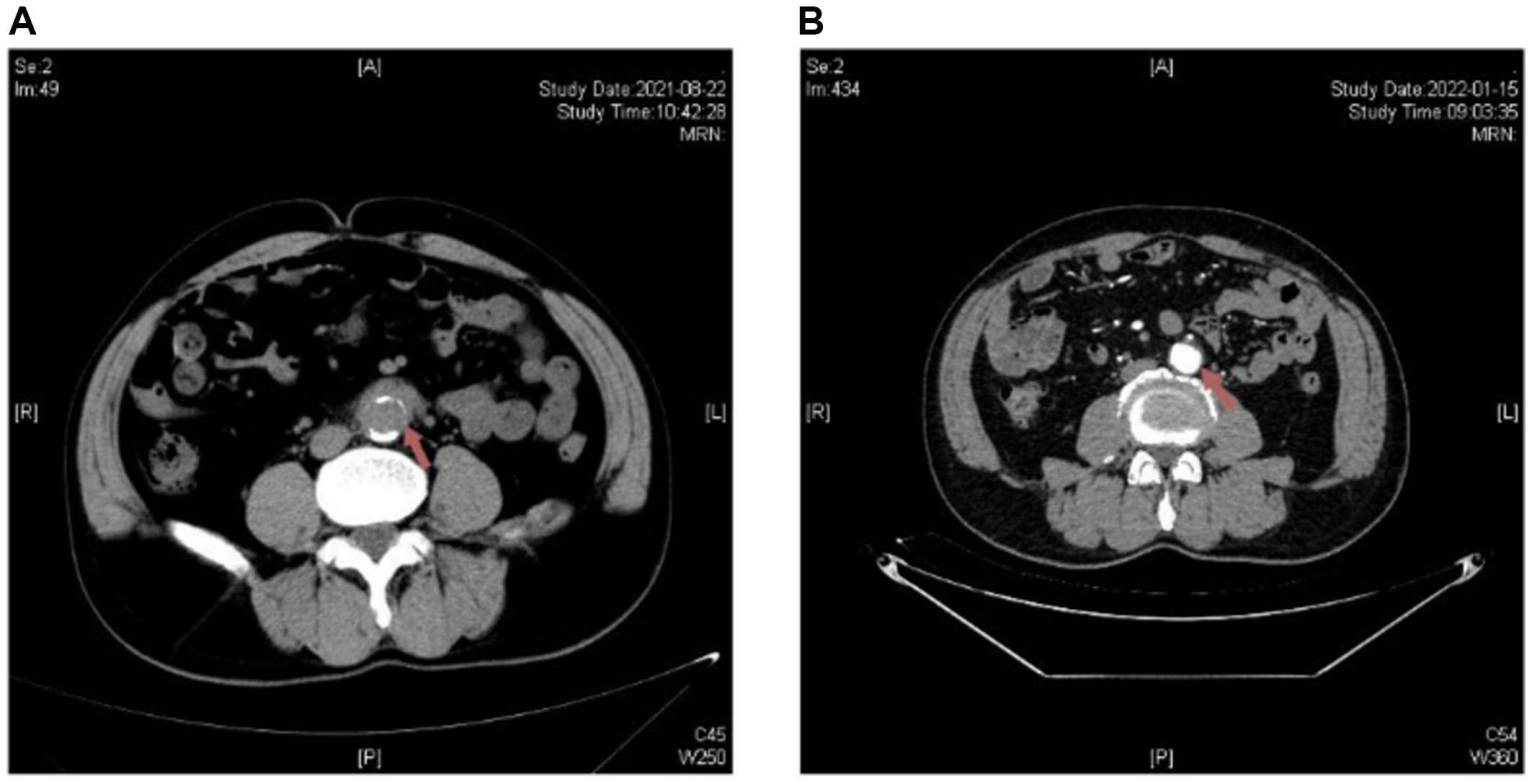
Radiological changes on abdominal aortic CTA before and after antibiotic treatment in Patient 3. **(A)** Upon admission: the distal end of the abdominal aorta is encircled by soft tissue density. **(B)** After treatment: the wall of the distal portion of the abdominal aorta shows uneven thickening, with significant improvement compared to the previous examination.

Given the small size of the aneurysm and the absence of rupture risk, early anti-Brucella therapy was initiated. Oral administration of rifampicin (600 mg once daily) and doxycycline (100 mg twice daily) was initiated, alongside intravenous levofloxacin (500 mg once daily). Due to the emergence of acute digestive symptoms associated with intravenous levofloxacin, it was replaced with intravenous ceftriaxone (2000 mg once daily). Encouragingly, the patient experienced relief from abdominal discomfort and pain after the first day of treatment. Considering the apparent clinical efficacy of antibiotic therapy and the small diameter of the aneurysm, a conservative treatment strategy was selected. The entire treatment course included a minimum of six weeks of antibiotic therapy and intensive clinical and radiological monitoring. At the follow-up clinic visit, 6 months after discharge, the patient reported no recurrence of abdominal pain. Moreover, there were no significant abnormalities observed in ESR or CRP, and the SAT titer gradually decreased to 1:200. CTA revealed a significant reduction in the size of the tissue surrounding the aneurysm (Figure 3).

### 3.4. Statistical results

Over the span of the last half-century, a cumulative count of 24 cases (documented across 23 distinct publications) depicting Brucella-IAAA have been documented within the repository of PubMed (18–40). The earliest recorded instance traces back to the year 1976. Pertinent data of relevance were meticulously extracted from each individual manuscript. This encompassed key variables such as the citation’s nomenclature, year of publication, gender distribution, age demographics, national origins, exposition to high-risk epidemiological factors, concurrent medical conditions, duration of patient-reported symptoms, taxonomical classification of the implicated Brucella species, outcomes of diagnostic assays encompassing blood culture, tissue culture, and serological evaluations, dimensions and morphological attributes of the abdominal aortic aneurysms, particulars concerning antibiotic therapeutic regimens (including specifics of duration and dosage), nuanced aspects of surgical interventions (inclusive of the surgical approach undertaken), and ultimate clinical outcomes as pertains to both follow-up assessments and emergent complications.

## 4. Discussion

Although the abdominal aorta is not conventionally recognized as a frequent site of Brucella infection, cases of Brucella-IAAA have been reported, representing an exceedingly rare occurrence, yet considered one of the most severe complications of brucellosis. Through analysis of the reported cases, we found that the age range of affected patients varies, with the oldest patient being 83 years old (28). The peak incidence of Brucella-IAAA occurs around 67 years of age, with a higher prevalence among males compared to females. The disease has been predominantly reported in Mediterranean countries, encompassing Portugal and Spain, while isolated cases have also been documented in China, Saudi Arabia, and the United States (22, 29–33, 36). Owing to the scarcity of evidence-based guidelines for this rare condition, limited experience is available to guide diagnosis, treatment, and prognostic evaluation, raising concerns about iatrogenic harm and accentuating the imperative for additional data. Consequently, further research is warranted to ascertain the benefits and risks associated with distinct diagnostic and therapeutic modalities for Brucella-IAAA. In this study, we present 3 cases of Brucella-IAAA and undertake an extensive review of pertinent literature to augment the clinical comprehension of this condition.

As previously discussed, Brucella-IAAA has predominantly been documented in Mediterranean countries. Nevertheless, despite being the most prevalent zoonotic disease worldwide, the reported cases in the Middle East and Latin America, two other significant endemic regions, are notably lower than in the Mediterranean region. This discrepancy could be attributed to limited access to medical resources and experience in diagnosing Brucella-IAAA in developing countries in these areas, leading to insufficient diagnostic proficiency and awareness, thereby resulting in a higher incidence of misdiagnosis and missed diagnosis. In fact, the inadequate understanding of this disease may also contribute to its underestimation on a global scale. In the existing literature, the clinical presentation of Brucella-IAAA has predominantly been characterized by atypical gastrointestinal symptoms, notably including abdominal pain and nausea (18–21). However, in cases where patients have relevant medical history, such as a previous Brucella infection or a history of high-risk exposure to the pathogen, or are experiencing unexplained fever, it is crucial to conduct more vigilant monitoring when these aforementioned symptoms are observed (41). In a case series involving 24 patients, 14 individuals tested positive in blood cultures (18, 19), while 9 showed positive results in tissue cultures. Remarkably, all patients within the last decade had either blood or tissue culture results, highlighting the evolving understanding of the disease among clinicians. The limited use of tissue culture is associated with the increasing adoption of intravascular therapy, emphasizing the advancements made in disease management. Among the patients, 22 individuals underwent at least one imaging examination, such as ultrasound, CTA or Digital Subtraction Angiography. These findings underscore the reliance on imaging protocols as the standard diagnostic approach for AAAs. Notably, many patients’ imaging exams revealed inflammation or ulceration surrounding the aneurysm, further supporting the diagnostic significance of imaging modalities, which could also provide supporting evidence and guidance for bacteriological diagnosis to a certain extent.

The World Health Organization has provided two primary diagnostic methods for Brucellosis in their guidelines. Bacteriological diagnosis involves staining smears, blood cultures, and tissue cultures, with the latter considered a diagnostic gold standard for Brucellosis. Serological tests, such as RBT, SAT (used alone or with 2-ME or DTT reduction), Coombs antiglobulin, CFT, and ELISA, are also recommended for diagnosis and have widespread use in clinical practice. However, detecting and diagnosing Brucella-IAAA can be challenging, as it may not exhibit distinctive symptoms. Diagnosis often requires a high level of suspicion, along with serologic testing, imaging studies like CTA, and tissue culture. A comprehensive medical history and thorough physical examination also play pivotal roles in identifying the underlying cause. In the cases we presented, all 3 patients exhibited predominantly atypical localized symptoms, notably encompassing abdominal pain, along with concomitant manifestations of nausea, vomiting, and lower limb swelling. Crucially, all patients had a characteristic medical history of close exposure to Brucella. The further CTA unveiled the presence of AAAs accompanied by diverse infiltrative shadows of varying densities. Regarding laboratory investigations, both RBPT and SAT yielded positive results in all 3 patients. However, only 1 patient had a positive blood culture, possibly attributed to the extended duration of the disease in the remaining 2 patients. In conclusion, early diagnosis is crucial in suspected cases of Brucella-IAAA. The diagnostic process should involve both pathogen detection and imaging techniques, given the absence of evident symptoms and signs in patients with Brucella-IAAAs. A comprehensive assessment, including medical history, close contact history, and imaging data, should be conducted. When Brucella infection is suspected, timely performance of serological tests and blood cultures is essential.

The management of Brucella-IAAA typically involves a combination of antibiotic therapy and surgical intervention (6). The antibiotics of choice for Brucella-IAAA are doxycycline and rifampin, which are administered for a minimum duration of 6 weeks (42). In the existing literature, most patients have received treatment with tetracyclines or cephalosporins antibiotics. Surgical or endovascular intervention may be required to address the aneurysm itself. In this literature review, a total of 13 patients underwent open surgery (19, 20, 26, 32–34, 36–39). Conversely, 9 patients underwent endovascular repair, in reported cases of Brucella-IAAA treated with EVAR, combination antibiotic therapy has yielded positive treatment outcomes (18, 21, 22, 24, 25, 27, 29, 32). There has been an increasing trend in the utilization of this technique over the past five years. Endovascular repair may be a favorable option, particularly for cases involving ruptured or high-risk AAA.

To sum up, when encountering suspected infectious aortic aneurysms, including those possibly caused by Brucella, a thorough review of medical history and imaging data is essential. This assessment should include aneurysm size, shape, and extent. Additionally, prompt empiric use of broad-spectrum antibiotics and blood cultures is advisable. European guidelines recommend complete repair for infectious abdominal aortic aneurysms (43). Although specific guidelines for Brucella-IAAAs are lacking, Although specific guidelines for Brucella-IAAAs are lacking, we believe that the former can be used as a reference. This entails scheduled aneurysm repair after achieving adequate infection control with antibiotics.

In our clinical practice, we have noticed that in hemodynamically stable patients with small, short-duration, mildly infected Brucella-IAAAs, and effective antibiotic control, a more conservative approach might be suitable. This approach involves close monitoring with serial imaging and scheduled repair. For patients at risk of rupture, immediate surgical intervention is essential, along with concurrent antibiotic administration. The choice of surgical approach depends on factors like aneurysm size, location, and the patient’s overall health. Traditionally, open surgery has been preferred, entailing aneurysm resection, extensive local debridement, and revascularization via extra-anatomical bypass or *in situ* reconstruction. Reported mortality rates after *in situ* grafting range from 5% to 49%, and after extra-anatomical bypass, from 24% to 50%. Infectious complications may occur in 0% to 20% after *in situ* grafts, with older data suggesting a similarly high complication rate after extra-anatomical bypass, including a concern for late aortic stump rupture in up to 20% (44, 45). However, the aneurysm’s location significantly impacts outcomes, and open surgery is not suitable for elderly or medically compromised patients due to its invasiveness. Despite the risk of graft infection, endovascular repair for infectious aortic aneurysms is increasingly common. Existing data shows a 55% 5-year survival rate with EVAR combined with long-term antibiotic therapy (46). The Swedish study also found that EVAR has significant early survival benefits in cases of severe infection or complications related to aneurysms (47). Therefore, based on our center’s experience and in the absence of clear EVAR contraindications, such as the lack of suitable stents or uncontrollable severe infection, endovascular therapy may be prioritized for Brucella-induced abdominal aortic aneurysms when feasible (48).

While the utilization of EVAR in Brucella-IAAAs is on the rise, it’s crucial not to underestimate the potential risk of graft infection associated with it. In addition to preoperative antibiotic administration and infection control, meticulous attention should be given to graft selection during the surgical procedure. Currently, there is no variance in infection susceptibility among different alloys used for grafts. Nevertheless, a preference should be given to grafts with excellent isolation properties and a minimal leak rate. Preoperative antibiotic soaking of the grafts has not demonstrated significant improvement in infection prevention. Currently, there are no available antibiotic-coated grafts, which could be a promising avenue for future research.

In cases of graft infection post-surgery, it is advisable to initiate an initial course of intensive antibiotic therapy for 4–6 weeks, with some experts advocating for antibiotic usage ranging from 3-6 months to even up to 1 year (49). For patients who cannot undergo open surgery, a lifelong regimen of antibiotic treatment is recommended. Furthermore, vigilant monitoring of imaging data is imperative to assess the extent of infection in proximity to the abdominal aortic aneurysm. If necessary, timely percutaneous drainage and flushing should be conducted. The EVAR procedure can also be considered a bridging treatment, with the option of subsequent open surgery if infection control is not optimal. In open surgery, *in situ* reconstruction entails the complete removal of infectious material and can be achieved using autologous veins, cryopreserved allografts, rifampin-coated or silver-coated synthetic grafts, as well as allografts.

In our center, we have observed that for patients with suspected Brucella-IAAA without clear indications for open surgery, endovascular repair can be considered as a treatment option. Certainly, it is crucial to promptly initiate appropriate antibiotic therapy. In critical conditions, such as suspected rupture or imminent rupture of the Brucella-IAAA, endovascular repair can be performed while simultaneously working on pathogen diagnosis. Broad-spectrum antibiotics should be administered empirically immediately after surgery, and adjustment to sensitive antibiotics should be made based on the results of blood culture. Among the 3 patients in this study, two individuals with surgical indications underwent endovascular repair, as one patient presented with a ruptured AAA and the other had a large aneurysm. The choice of endovascular repair over open surgery was primarily based on its favorable therapeutic outcomes and prognosis for ruptured AAA. Additionally, one patient exhibited abnormal immune indicators before surgery, and endovascular repair was deemed advantageous due to its reduced surgical trauma and shorter hospital stay, particularly for patients with potential immune abnormalities. Following endovascular repair, both patients experienced immediate relief from abdominal pain symptoms. However, owing to the ongoing Brucella infection, they exhibited fluctuating body temperatures and recurring symptoms. These symptoms subsided rapidly upon the administration of specific antibiotics. The remaining patient opted for a conservative treatment plan involving antibiotics and regular follow-up due to his smaller aneurysms. This treatment approach was supported by the patient and his families. All 3 patients commenced drug treatment upon suspected diagnosis, as antibiotics constitute the most effective strategy for managing Brucella infections. The two patients who underwent surgery received a six-month regimen of minocycline, rifampin, and ceftriaxone/levofloxacin, while the non-surgical patients received doxycycline, rifampin, and ceftriaxone treatment. Although both regimens involve tetracycline antibiotics, it was observed that doxycycline exhibits a higher affinity for calcium and may yield better therapeutic effects when combined with endovascular stent grafts, while minocycline lacks stent affinity (50, 51). Therefore, different tetracycline-class drugs were selected for individual patients, all of whom achieved favorable therapeutic outcomes.

Overall, a multidisciplinary approach involving early diagnosis, appropriate treatment selection, and careful management is crucial for optimal outcomes in patients with suspected or confirmed Brucella-IAAA.

The follow-up period for AAA is considered a long-term process, typically extending for at least one year or more (52). Given the relatively recent occurrence of the cases included in this study, it is currently not possible to provide predictions regarding the long-term condition of the 3 patients. Moreover, it is important to note that the oldest patient among the reported cases in our center is 67 years old, which can be considered relatively young. Consequently, further research is required to enhance our understanding and treatment experience in managing patients aged over 70 years. This limitation underscores the need for additional investigations in this area.

In conclusion, this study presents 3 cases of Brucella-IAAA and reviews an additional 24 cases to provide a comprehensive understanding of the clinical characteristics and treatment options available. The findings serve as a scientific basis for accurate diagnosis and the development of effective treatment strategies for Brucella-IAAA. Currently, there is no standardized approach for the diagnosis and treatment of this condition. However, early initiation of diagnosis and treatment for suspected cases of Brucella-IAAA is crucial to enable timely intervention, which is vital in slowing disease progression and reducing surgical risks.

## Data availability statement

The original contributions presented in the study are included in the article/supplementary material, further inquiries can be directed to the corresponding authors.

## Ethics statement

Written informed consent was obtained from the individual(s) for the publication of any potentially identifiable images or data included in this article. This study has obtained approval from the Ethics Committee of Qingdao University Affiliated Hospital.

## Author contributions

HM: Data curation, Data curation, Writing – original draft. YY: Investigation, Writing – review & editing. HL: Data curation, Investigation, Writing – original draft. XS: Conceptualization, Writing – original draft. YL: Writing – review & editing. MG: Writing – review & editing.
